# Supplementary material for: Drivers of ESBL-producing Escherichia coli dynamics in calf fattening farms: A modelling study
Source: One Health. 2021 Mar 18;12:100238. doi: 10.1016/j.onehlt.2021.100238 (PMC8022845; doi:10.1016/j.onehlt.2021.100238)
Supplement: Supplementary file 1 — Supplementary material [file mmc1.pdf]

# Supplementary material

# Drivers of ESBL-producing *Escherichia coli* dynamics in calf fattening farms: a modelling study

**J. Bastard, M. Haenni, E. Gay, P. Glaser, J.Y. Madec, L. Temime, L. Opatowski**

## SM1. Longitudinal data of ESBL-EC carriage in calves

***Table S1. ESBL-EC carriage across time in calves.*** *The last sampling day was day 161 in farms A and B, and day 147 in farm C. ESBL-EC positive (resp. negative) samples are represented as 1 (resp. 0).*

| | | | Sampling days | | | | | | | | | | | |
|---|---|---|---|---|---|---|---|---|---|---|---|---|---|---|
| **Farm** | **Pen** | **Calf** | **7** | **21** | **35** | **49** | **63** | **77** | **91** | **106** | **119** | **133** | **147** | **161** |
| **A** | **A-1** | **1** | 1 | 1 | 1 | 1 | 1 | 0 | 0 | 0 | 0 | 0 | 0 | 0 |
| **A** | **A-1** | **2** | 1 | 1 | 1 | 1 | 0 | 0 | 0 | 0 | 0 | 0 | 0 | 0 |
| **A** | **A-1** | **3** | 1 | 1 | 1 | 1 | 1 | 1 | 0 | 0 | 0 | 0 | 0 | 1 |
| **A** | **A-1** | **4** | 1 | 1 | 1 | 1 | 1 | 0 | 0 | 0 | 0 | 0 | 0 | 0 |
| **A** | **A-1** | **5** | 1 | 1 | 1 | 1 | 0 | 0 | 0 | 0 | 0 | 0 | 0 | 0 |
| **A** | **A-2** | **6** | 1 | 1 | 1 | 1 | 1 | 0 | 0 | 0 | 0 | 0 | 0 | 0 |
| **A** | **A-2** | **7** | 1 | 1 | 1 | 1 | 1 | 0 | 0 | 0 | 0 | 0 | 0 | 0 |
| **A** | **A-2** | **8** | 1 | 1 | 1 | 1 | 1 | 1 | 0 | 0 | 0 | 0 | 0 | 0 |
| **A** | **A-2** | **9** | 1 | 1 | 1 | 1 | 1 | 0 | 0 | 0 | 0 | 0 | 0 | 0 |
| **A** | **A-2** | **10** | 1 | 1 | 1 | 1 | 1 | 1 | 0 | 0 | 0 | 0 | 0 | 0 |
| **A** | **A-3** | **11** | 1 | 1 | 1 | 1 | 1 | 1 | 0 | 0 | 1 | 0 | 1 | 0 |
| **A** | **A-3** | **12** | 1 | 1 | 1 | 1 | 1 | 0 | 0 | 0 | 0 | 0 | 0 | 0 |
| **A** | **A-3** | **13** | 1 | 1 | 1 | 1 | 1 | 1 | 1 | 0 | 0 | 0 | 1 | 0 |
| **A** | **A-3** | **14** | 1 | 1 | 1 | 1 | 1 | 0 | 0 | 0 | 0 | 0 | 0 | 0 |
| **A** | **A-3** | **15** | 1 | 1 | 1 | 1 | 1 | 1 | 0 | 0 | 0 | 0 | 0 | 0 |
| **B** | **B-1** | **16** | 0 | 0 | 0 | 0 | 0 | 0 | 0 | 0 | 0 | 0 | 0 | 0 |
| **B** | **B-1** | **17** | 0 | 0 | 0 | 0 | 0 | 0 | 0 | 0 | 0 | 0 | 0 | 0 |
| **B** | **B-1** | **18** | 0 | 0 | 0 | 0 | 0 | 0 | 0 | 0 | 0 | 0 | 0 | 0 |
| **B** | **B-1** | **19** | 0 | 0 | 0 | 0 | 0 | 0 | 0 | 0 | 0 | 0 | 0 | 0 |
| **B** | **B-1** | **20** | 0 | 0 | 0 | 0 | 0 | 0 | 0 | 0 | 0 | 0 | 0 | 0 |

| | | | | | | | | | | | | | | |
|---|---|---|---|---|---|---|---|---|---|---|---|---|---|---|
| **B** | **B-2** | **21** | 1 | 0 | 0 | 1 | 0 | 0 | 0 | 0 | 0 | 0 | 0 | 0 |
| **B** | **B-2** | **22** | 1 | 0 | 0 | 0 | 0 | 0 | 0 | 0 | 0 | 0 | 0 | 0 |
| **B** | **B-2** | **23** | 1 | 1 | 1 | 0 | 0 | 0 | 0 | 0 | 0 | 0 | 0 | 0 |
| **B** | **B-2** | **24** | 1 | 0 | 0 | 0 | 0 | 0 | 0 | 0 | 0 | 0 | 0 | 0 |
| **B** | **B-2** | **25** | 1 | 1 | 0 | 0 | 0 | 0 | 0 | 0 | 0 | 0 | 0 | 0 |
| **B** | **B-3** | **26** | 1 | 0 | 0 | 0 | 0 | 0 | 0 | 0 | 0 | 0 | 0 | 0 |
| **B** | **B-3** | **27** | 1 | 0 | 1 | 0 | 0 | 0 | 0 | 0 | 0 | 0 | 0 | 0 |
| **B** | **B-3** | **28** | 1 | 0 | 0 | 0 | 0 | 0 | 0 | 0 | 0 | 0 | 0 | 0 |
| **B** | **B-3** | **29** | 1 | 0 | 0 | 0 | 0 | 0 | 0 | 0 | 0 | 0 | 0 | 0 |
| **B** | **B-3** | **30** | 1 | 0 | 0 | 0 | 0 | 0 | 0 | 0 | 0 | 0 | 0 | 0 |
| **C** | **C-1** | **31** | 0 | 1 | 1 | 0 | 0 | 0 | 0 | 0 | 1 | 1 | 1 | / |
| **C** | **C-1** | **32*** | 0 | 1 | 1 | 0 | / | / | / | / | / | / | / | / |
| **C** | **C-1** | **33** | 0 | 0 | 1 | 0 | 0 | 0 | 0 | 0 | 1 | 1 | 0 | / |
| **C** | **C-1** | **34** | 0 | 0 | 1 | 0 | 1 | 0 | 0 | 0 | 1 | 0 | 0 | / |
| **C** | **C-2** | **35** | 1 | 1 | 1 | 0 | 1 | 0 | 0 | 0 | 1 | 1 | 1 | / |
| **C** | **C-2** | **36** | 1 | 1 | 1 | 0 | 0 | 0 | 1 | 0 | 1 | 0 | 0 | / |
| **C** | **C-2** | **37** | 1 | 1 | 1 | 0 | 0 | 0 | 0 | 0 | 1 | 1 | 0 | / |
| **C** | **C-2** | **38** | 1 | 1 | 1 | 0 | 0 | 0 | 0 | 1 | 1 | 1 | 1 | / |
| **C** | **C-2** | **39** | 1 | 0 | 1 | 0 | 1 | 0 | 0 | 1 | 1 | 1 | 0 | / |
| **C** | **C-3** | **40** | 1 | 1 | 1 | 1 | 1 | 0 | 0 | 0 | 1 | 1 | 0 | / |
| **C** | **C-3** | **41** | 1 | 0 | 1 | 0 | 0 | 0 | 0 | 0 | 1 | 1 | 0 | / |
| **C** | **C-3** | **42** | 1 | 0 | 1 | 0 | 1 | 0 | 0 | 0 | 1 | 1 | 0 | / |
| **C** | **C-3** | **43** | 1 | 0 | 1 | 0 | 1 | 0 | 0 | 1 | 0 | 1 | 0 | / |
| **C** | **C-3** | **44** | 1 | 0 | 1 | 1 | 1 | 1 | 0 | 1 | 1 | 1 | 0 | / |
| **C** | **C-3** | **45** | 1 | 0 | 1 | 0 | 0 | 0 | 0 | 0 | 1 | 1 | 1 | / |

* Calf #32 died between the $4^{th}$ and the $5^{th}$ sampling days.

**SM2. Details on the models**

A schematic representation of the model is presented in Figure 1 of the main text. We assumed the dynamics of ESBL-EC were independent of other *E. coli*. At time *t*, each ESBL-EC negative calf could acquire an ESBL-EC with a probability

$$P = 1\text{-}e^{-A(t)}$$

where $A(t)$ was the acquisition rate, which formula depended on the model variant as detailed in Table S2. $A(t)$ was generally defined as:

$$A(t) = [A_{trans}(t) + A_{spor}(t)] \times \lambda_a(t)$$

with, at time *t*, $A_{trans}(t)$ the acquisition term related to between-calves transmission, $A_{spor}(t)$ the term related to sporadic contaminations and $\lambda_a(t)$ the multiplicative effect related to AMU.

*Transmission model*. Transmission was assumed to occur either homogeneously between calves of the same farm *F*, with rate $\beta_f^F$, or between calves depending on their attributed pen, assuming two transmission rates, within ($\beta_w^F$) and between ($\beta_b^F$) pens of a farm *F*. The transmission rate between individual pens was assumed to be the same as between collective pens. Transmission was supposed to be proportional to the proportion of calves carrying ESBL-EC. As a null hypothesis, we also investigated models, which did not include any transmission between calves, but instead a constant, farm-specific ESBL-EC acquisition rate $\beta_0^F$. Therefore, at time *t*:

$A_{trans}(t) = \beta_f^F \left(\frac{I}{S+I}\right)_{F,\, t-1}$ for homogeneous mixing (models 1a, 3a, 5a, 1b, 3b and 5b)

$= \beta_w^F \left(\frac{I}{S+I}\right)_{in\ pen,\, t-1} + \beta_b^F \left(\frac{I}{S+I}\right)_{out\ pen,\, t-1}$ for pen-specific mixing (models 2a, 4a, 6a, 2b, 4b and 6b)

$= \beta_0^F$ for the baseline acquisition rate (models, 0a, 7a, 8a, 0b, 7b and 8b)

where *I* was the number of ESBL-EC positive calves in the farm (within the pen, outside the pen or globally in the farm) and *S* the number of ESBL-EC negative calves.

*Sporadic contamination*. We also assumed that sporadic contamination could occur, depicting the possible acquisition of ESBL-EC by the calves on farm *F* on some specific days from another (unknown) source other than the colonised calves. To model this process, we assumed that sporadic contamination events in a given farm increased the acquisition rate $A(t)$ of all calves on the farm on the day of contamination by a factor $\mu$. We estimated $N^F$, the number of days with contamination events that occurred across the follow up, and $D^F$, the specific dates of contamination. This mechanism was activated in models 0a to 8a.

We defined: $A_{spor}(t) = \mu$ if $t \in D^F$ (= 0 otherwise)

*Clearance.* ESBL-EC positive calves could clear carriage with a probability $1\text{-}e^{-C(t)}$, where $C(t)$ was the clearance rate at time *t*. In the baseline model, we assumed a natural clearance rate, $\nu_0$ (inverse of the baseline carriage duration).

*Impact of antibiotics.* We modelled the possible effect of AMU through two mechanisms. On the one hand, we assumed that AMU could affect the acquisition rate *A(t)*. For a given antibiotic class *i*, this was modelled by a multiplicative factor $\alpha_{a,i}$. After the end of exposure, this effect was supposed to persist [1], but decrease exponentially [2] with a rate $\tau$, common to all antibiotic classes, until it reaches a saturation at the value of 1 (see SM7 of this Supplementary material for illustration). This mechanism was active for models 3a, 4a, 7a, 3b, 4b and 7b (Table 1, main text).

Therefore, in these models, at time *t*,

$$\lambda_a(t) = \prod_i \alpha_{a,i}^{max(0\,;\,1-\tau Ei(t))} \qquad \text{(= 1 otherwise)}$$

where $E_i(t)$ was the number of days since the end of the last exposure of the calf to antibiotic class *i*. Therefore, if a calf was exposed to antibiotic class *i* on day t, $E_i(t)$=0. During model fitting, the antimicrobial exposure considered was only that observed in the longitudinal study.

On the other hand, we assumed that antibiotics could impact clearance by modulating the baseline clearance rate $v_0$ as follows (activated in models 5a, 6a, 8a, 5b, 6b and 8b):

$$C(t) = \nu_0 . \lambda_c(t) = \nu_0 . \prod_i \alpha_{c,i}^{max(0\,;\,1-\tau Ei(t))} \qquad \text{(= } \nu_0 \text{ otherwise)}$$

where $E_i(t)$ and $\tau$ were as defined above, and $\alpha_{c,i}$ was a multiplicative factor analogous to $\alpha_{a,i}$, representing the effect of exposure to antibiotic class *i* on the clearance rate *C(t).*

***Table S2. Expressions of the acquisition rate* A(t) *and clearance rate* C(t)*, for the considered models.***

$\lambda_a(t) = \prod_i \alpha_{a,i}^{\max(0\,;\,1-\tau Ei(t))}$ *and* $\lambda_c(t) = \prod_i \alpha_{c,i}^{\max(0\,;\,1-\tau Ei(t))}$. *Parameter* $\alpha_{a,i}$ *(resp.* $\alpha_{c,i}$*) corresponds to the effect of antimicrobial class* i *on acquisition (resp. clearance).* $E_i(t)$ *is the number of days since the end of the last exposure to antibiotic class* i*, while* τ *is the rate of the exponential decay of the antimicrobial's effect over time, after the end of exposure.* I *(resp.* S*) is the number of ESBL-EC positive (resp. negative) calves, while* t *represents the day and* F *is either farm A, B or C.*

| Model | Acquisition rate *A(t)* | Clearance rate *C(t)* |
|---|---|---|
| 0a | $A_{spor}(t) + \beta_0^F$ | $\nu_0$ |
| 1a | $A_{spor}(t) + \beta_f^F \left(\frac{I}{S+I}\right)_{F,\ t-1}$ | $\nu_0$ |
| 2a | $A_{spor}(t) + \beta_w^F \left(\frac{I}{S+I}\right)_{in\ pen,\ t-1} + \beta_b^F \left(\frac{I}{S+I}\right)_{out\ pen,\ t-1}$ | $\nu_0$ |
| 3a | $[A_{spor}(t) + \beta_f^F \left(\frac{I}{S+I}\right)_{F,\ t-1}] \times \lambda_a(t)$ | $\nu_0$ |
| 4a | $[A_{spor}(t) + \beta_w^F \left(\frac{I}{S+I}\right)_{in\ pen,\ t-1} + \beta_b^F \left(\frac{I}{S+I}\right)_{out\ pen,\ t-1}] \times \lambda_a(t)$ | $\nu_0$ |
| 5a | $A_{spor}(t) + \beta_f^F \left(\frac{I}{S+I}\right)_{F,\ t-1}$ | $\nu_0 \times \lambda_c(t)$ |
| 6a | $A_{spor}(t) + \beta_w^F \left(\frac{I}{S+I}\right)_{in\ pen,\ t-1} + \beta_b^F \left(\frac{I}{S+I}\right)_{out\ pen,\ t-1}$ | $\nu_0 \times \lambda_c(t)$ |
| 7a | $[A_{spor}(t) + \beta_0^F] \times \lambda_a(t)$ | $\nu_0$ |
| 8a | $A_{spor}(t) + \beta_0^F$ | $\nu_0 \times \lambda_c(t)$ |
| 0b | $\beta_0^F$ | $\nu_0$ |
| 1b | $\beta_f^F \left(\frac{I}{S+I}\right)_{F,\ t-1}$ | $\nu_0$ |
| 2b | $\beta_w^F \left(\frac{I}{S+I}\right)_{in\ pen,\ t-1} + \beta_b^F \left(\frac{I}{S+I}\right)_{out\ pen,\ t-1}$ | $\nu_0$ |
| 3b | $[\beta_f^F \left(\frac{I}{S+I}\right)_{F,\ t-1}] \times \lambda_a(t)$ | $\nu_0$ |
| 4b | $[\beta_w^F \left(\frac{I}{S+I}\right)_{in\ pen,\ t-1} + \beta_b^F \left(\frac{I}{S+I}\right)_{out\ pen,\ t-1}] \times \lambda_a(t)$ | $\nu_0$ |
| 5b | $\beta_f^F \left(\frac{I}{S+I}\right)_{F,\ t-1}$ | $\nu_0 \times \lambda_c(t)$ |
| 6b | $\beta_w^F \left(\frac{I}{S+I}\right)_{in\ pen,\ t-1} + \beta_b^F \left(\frac{I}{S+I}\right)_{out\ pen,\ t-1}$ | $\nu_0 \times \lambda_c(t)$ |
| 7b | $\beta_0^F \times \lambda_a(t)$ | $\nu_0$ |
| 8b | $\beta_0^F$ | $\nu_0 \times \lambda_c(t)$ |

**SM3. Code sharing and details on modelling assumptions and on the estimation using MCMC**

We explored the space of possibilities for acquisition and clearance dates for each individual using data augmentation [3, 4]. Based on previous estimates of the time to clearance of ESBL-producing *Enterobacterales* [5-7], we assumed that, for a given calf, if two consecutive samples were positive (resp. negative) for ESBL-EC carriage, the calf was ESBL-EC positive (resp. negative) everyday between the two sampling dates.

Model parameters were estimated in a Bayesian framework, using a Markov Chain Monte Carlo (MCMC) algorithm, implemented with the R package *rjags* [8]. For each model variant, 3 chains were run for 6,000 iterations each. Depending on the model variant, from 2,000 to 10,000 values were discarded as burn-in, such that convergence of the Markov chains was reached. We sampled every 40th value because this was enough to avoid autocorrelation within the Markov chains. The effective sample size was at least 328 for posteriors of each model (953 for model 5a, the model selected). A Gelman-Rubin statistic below 1.1 for all parameters was considered to indicate that between-chain variance was low enough compared to the within-chain variance [9]. The exception was model 4 where, if convergence of each chain was reached, the between-chain variance was still high, no matter the length of the burn-in. Non-informative uniform priors were used for all parameters (Table 2, main text). The 18 models were compared using the Deviance Information Criterion (DIC) [10]. The model for which the DIC was the lowest was selected as the best model (Table S3).

All analyses of this paper, including models estimation and simulation, were performed using R version 3.6.1. The code is available on the following link:

https://github.com/JonathanBas/model_ESBL_calves.

## SM4. Antimicrobial use observed in farms

In our study, no individual treatment was observed: antibiotics were always administered to all calves on each farm simultaneously.

On farm A, five treatments were observed: colistin and sulfonamides between days 1 and 10, tetracycline between days 11 and 20, doxycycline on day 53, and amoxicillin on day 136 (Figure 2, main text). On farm B, calves were treated with doxycycline and erythromycin on day 26, tetracycline on day 90, and amoxicillin on day 101, resulting in four one-day treatments. On farm C, calves were exposed to five treatments: sulfonamide-trimethoprim combination between days 3 and 8, followed by tetracycline between days 10 and 16, doxycycline between days 20 and 24, spiramycin between days 25 and 27, and tetracycline between days 80 and 82.

## SM5. DIC of models

***Table S3. Deviance Information Criterion (DIC) of models.*** *Model 5a presented the lowest DIC.*

| **Model** | **0a** | **1a** | **2a** | **3a** | **4a** | **5a** | **6a** | **7a** | **8a** |
|---|---|---|---|---|---|---|---|---|---|
| **DIC** | 223.6 | 217.7 | 225.5 | 224.0 | 246.2 | 196.3 | 212.0 | 230.3 | 201.9 |
| **Model** | **0b** | **1b** | **2b** | **3b** | **4b** | **5b** | **6b** | **7b** | **8b** |
| **DIC** | 256.1 | 238.5 | 248.0 | 236.6 | 250.9 | 219.1 | 232.6 | 251.6 | 239.2 |

## SM6. Posterior distributions in model 5a

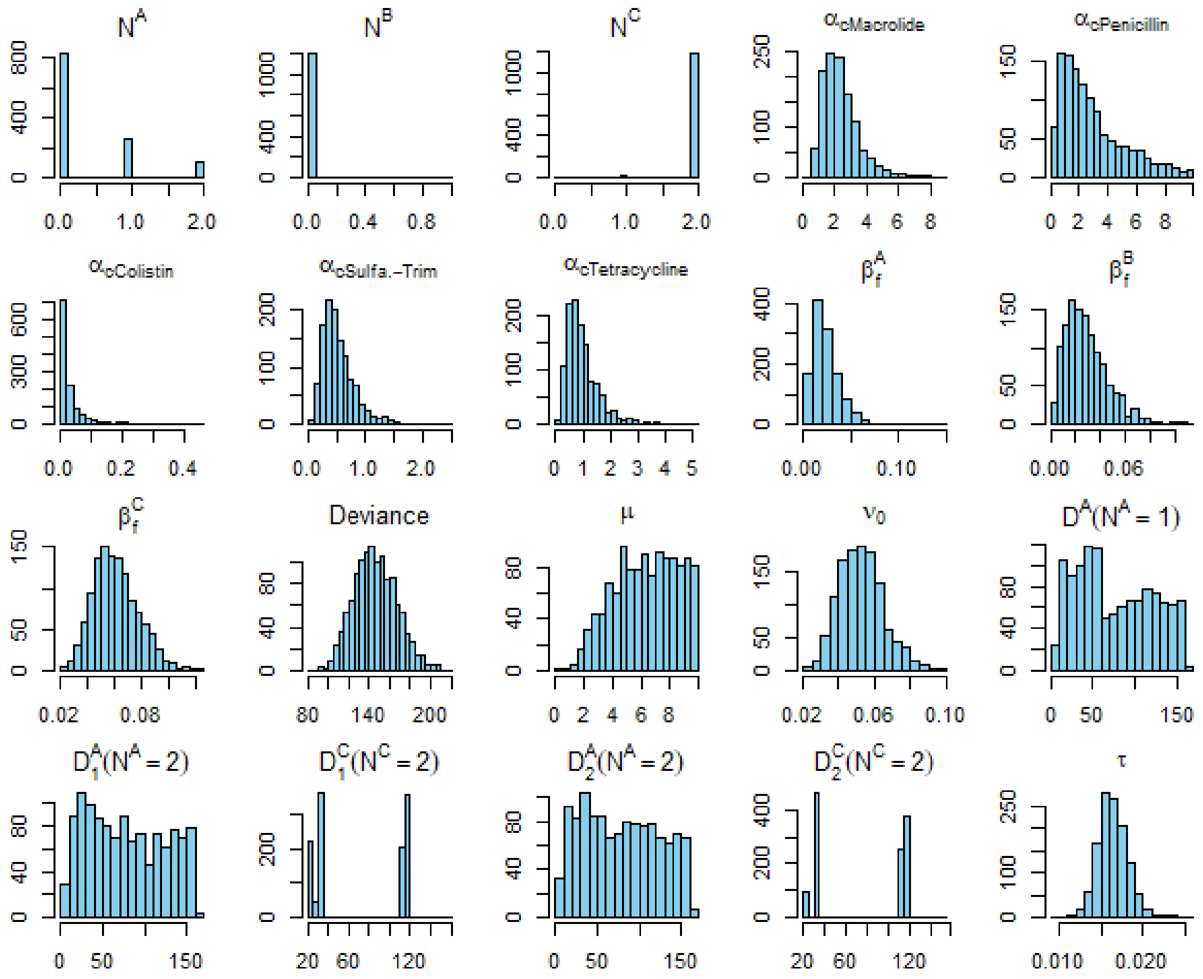


***Figure S1. Posterior distributions for the best model, model 5a.*** *Parameters (and their prior distributions) are defined in Table 2 of the main text. $D^F(N^F=1)$ is the date of occurrence of the sporadic contamination event in farm F when $N^F$ (number of sporadic contamination events in farm F) is estimated to be 1. $D_1^F(N^F=2)$ and $D_2^F(N^F=2)$ are the dates of the two sporadic contamination events in farm F when $N^F$ is estimated to be 2. The posterior distributions of the dates of sporadic contamination events in farm A,* i.e. *$D^A(N^A=1)$, $D_1^A(N^A=2)$ and $D_2^A(N^A=2)$, were close to their prior distributions, because a majority (69.3%) of $N^A$ posterior samples were 0 (see Table 3 of the main text). Therefore, these dates parameters were hardly affected by the data. Similarly, the posterior distributions of $D^B(N^B=1)$, $D_1^B(N^B=2)$, $D_2^B(N^B=2)$ corresponded to their prior distributions and were not displayed. On the contrary, these dates were clearly estimated around days 22, 35, 114 and 117 for farm C.*

**SM7. Evolution of the clearance rate *C(t)* after the end of colistin use**

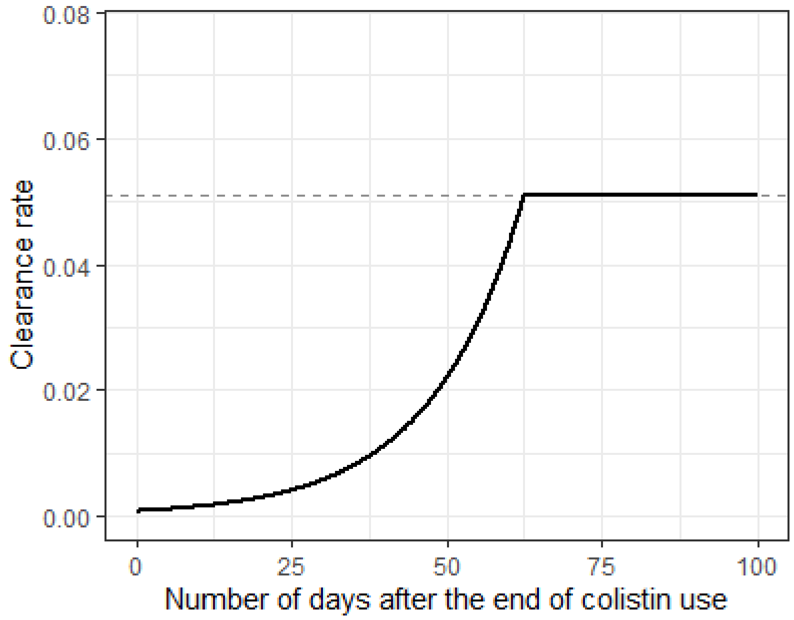


***Figure S2.*** *Evolution of the clearance rate after the end of colistin use (solid line), using median estimated values of parameters in Model 5a (Table 3, main text). After a delay, the clearance rate* C(t) *equals* $v_0$ *(dashed line).*

## SM8. Separate fits of each farm using model 5a

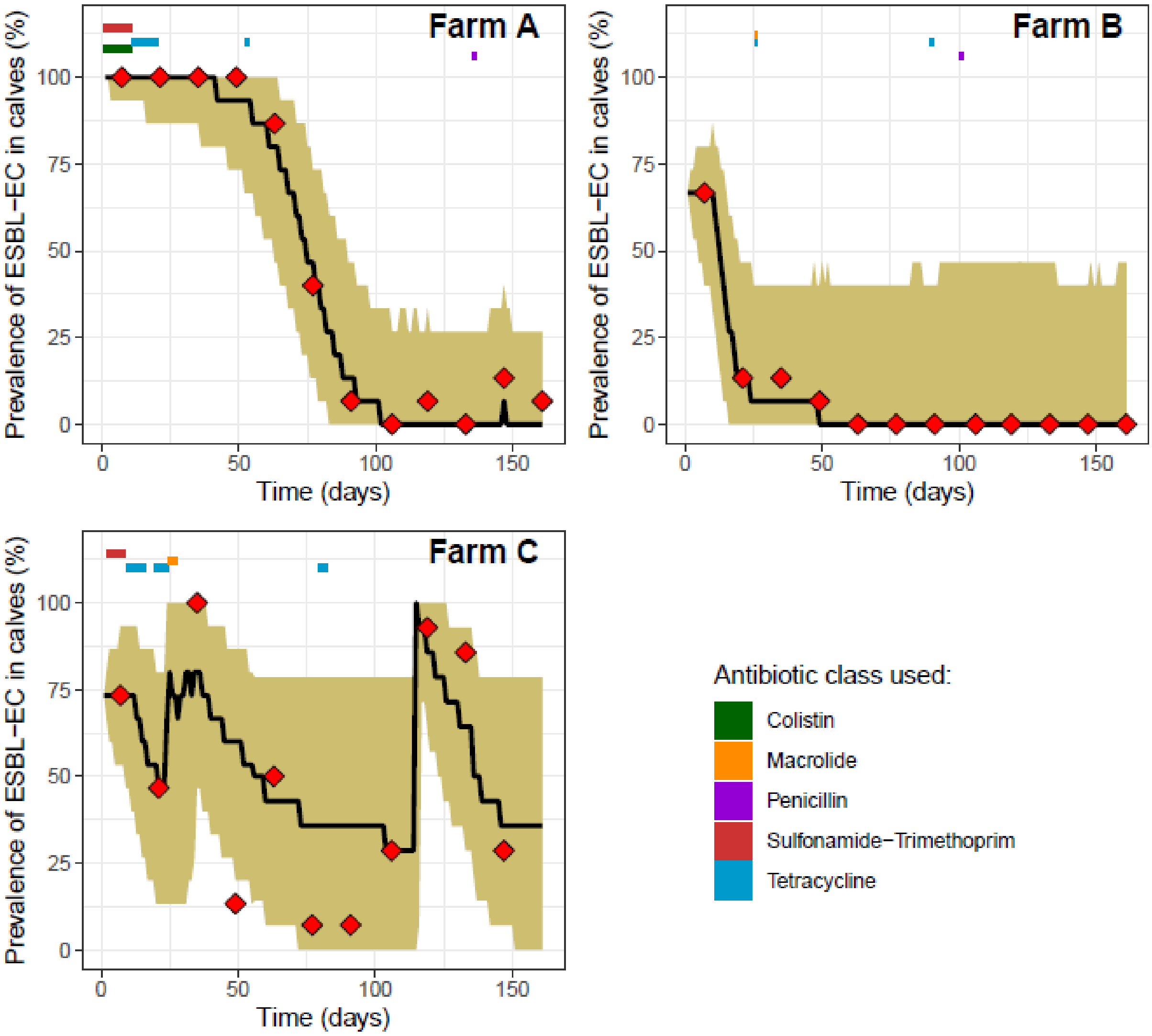


***Figure S3.*** *Observed (red dots), median predicted ESBL-EC prevalence (black line) and 95% prediction interval using model 5a for each farm separately (5,000 repetitions of the model). Observed periods of antimicrobial use are represented for each antibiotic class.*

## SM9. Sensitivity analysis on the farm selected for the simulations study

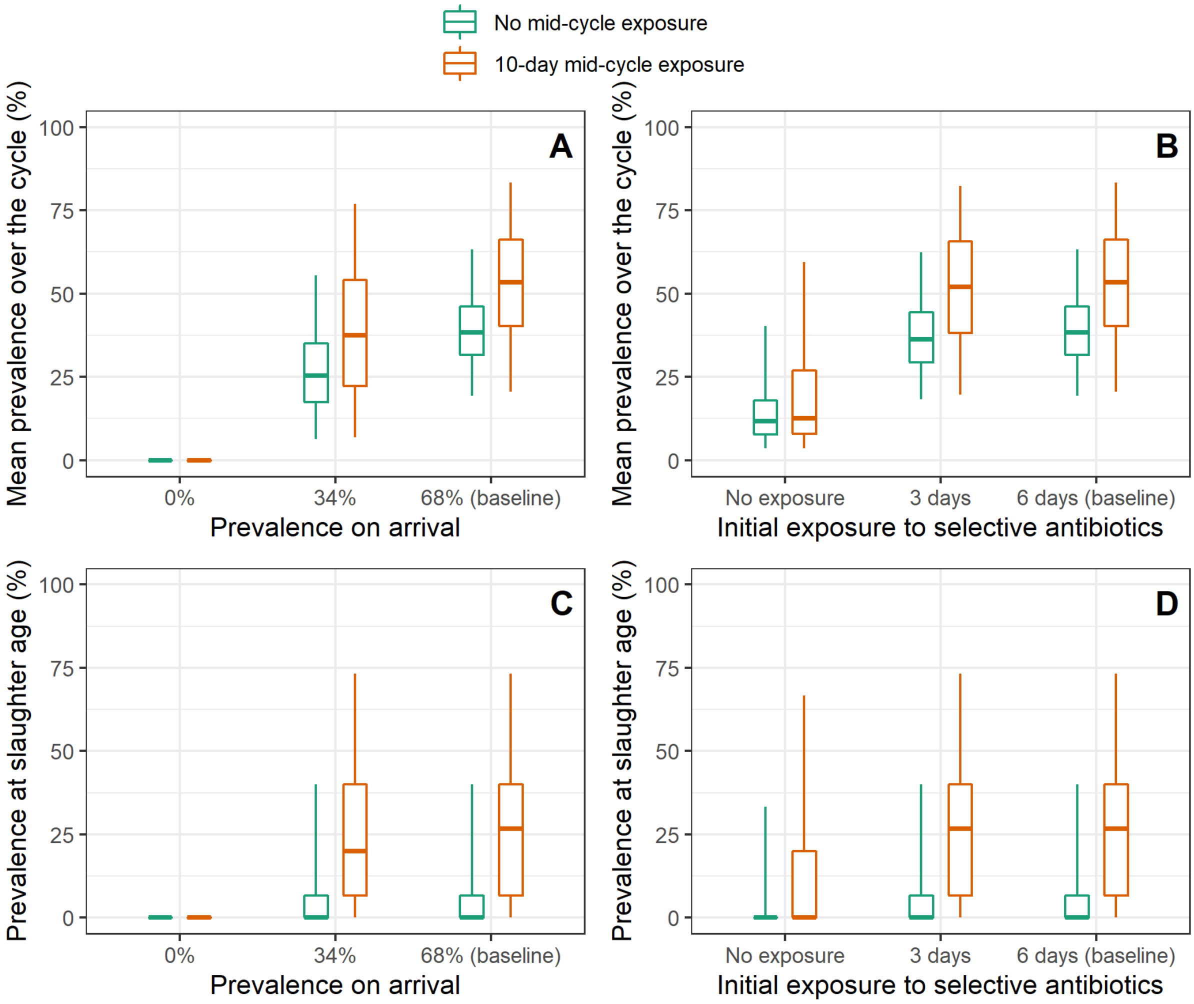


***Figure S4. Simulations of ESBL-EC mitigation strategies in farm B.*** *Mean ESBL-EC prevalence over the production cycle (panels A and B) and prevalence at slaughter age (panels C and D) predicted in farm B by model 5a (5,000 repetitions of the model), when ESBL-EC prevalence on arrival (panels A and C) and the duration of the initial antibiotic exposure (panels B and D) are changed from their baseline values. Scenarios without (turquoise) or with (orange) a 10-day antibiotic exposure in the middle of the fattening cycle (between days 81 and 90) are explored. Values represented by the boxes are the predicted median, and the 50% and 95% prediction intervals.*

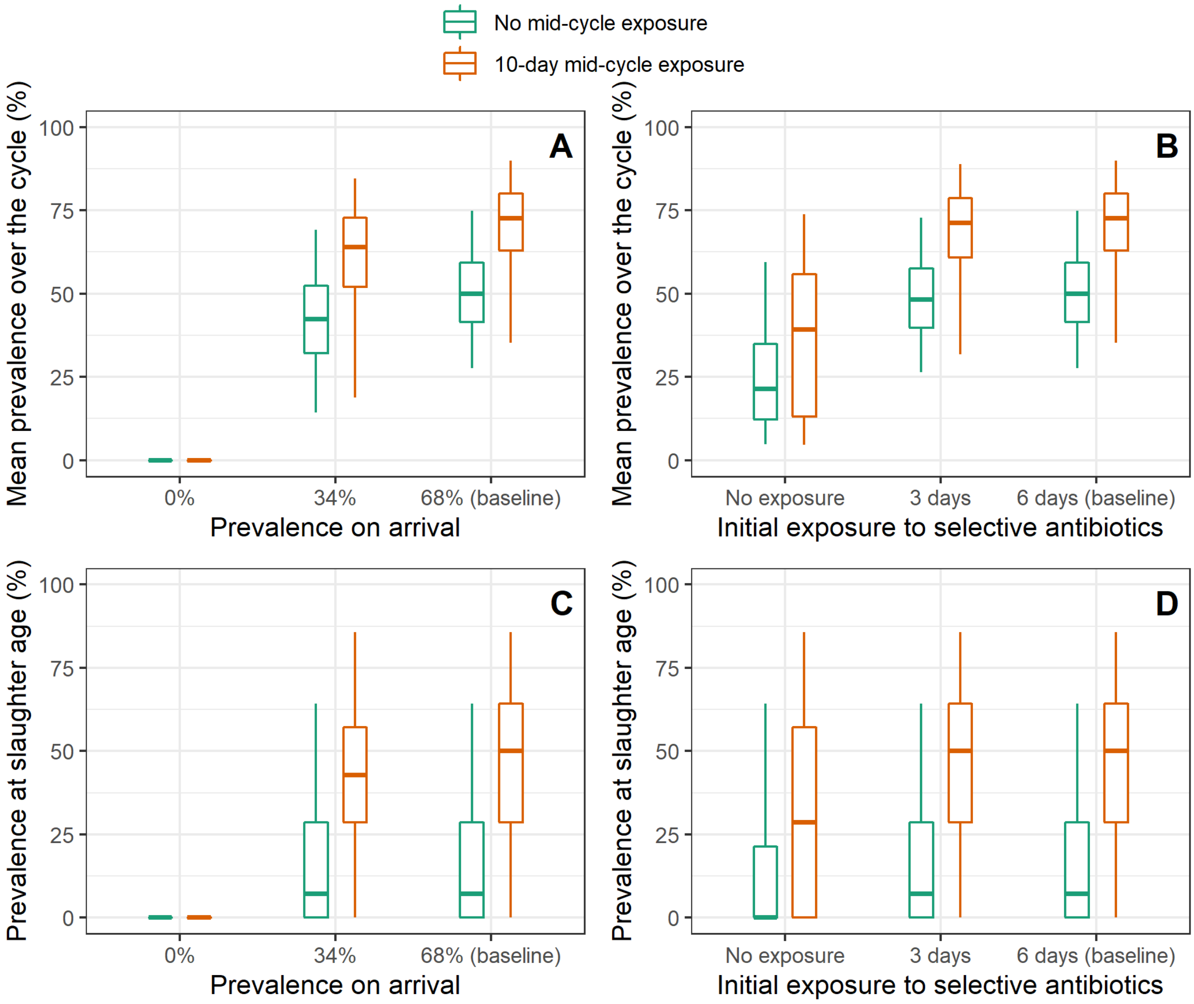


***Figure S5. Simulations of ESBL-EC mitigation strategies in farm C.*** *Mean ESBL-EC prevalence over the production cycle (panels A and B) and prevalence at slaughter age (panels C and D) predicted in farm C by model 5a (5,000 repetitions of the model), when ESBL-EC prevalence on arrival (panels A and C) and the duration of the initial antibiotic exposure (panels B and D) are changed from their baseline values. Scenarios without (turquoise) or with (orange) a 10-day antibiotic exposure in the middle of the fattening cycle (between days 81 and 90) are explored. Values represented by the boxes are the predicted median, and the 50% and 95% prediction intervals.*

# References of the Supplementary material


[1] S. Caballero, S. Kim, R.A. Carter, I.M. Leiner, B. Sušac, L. Miller, et al. Cooperating Commensals Restore Colonization Resistance to Vancomycin-Resistant Enterococcus faecium. Cell Host Microbe. 2017;21(5):592-602.e4.

[2] E.N. Abatih, L. Alban, A.K. Ersbøll, D.M. Lo Fo Wong. Impact of antimicrobial usage on the transmission dynamics of antimicrobial resistant bacteria among pigs. Journal of Theoretical Biology. 2009;256(4):561-73.

[3] S. Cauchemez, L. Temime, A.-J. Valleron, E. Varon, G. Thomas, D. Guillemot, et al. S. pneumoniae transmission according to inclusion in conjugate vaccines: Bayesian analysis of a longitudinal follow-up in schools. BMC Infect Dis. 2006;6:14-.

[4] B.S. Cooper, G.F. Medley, S.J. Bradley, G.M. Scott. An Augmented Data Method for the Analysis of Nosocomial Infection Data. American Journal of Epidemiology. 2008;168(5):548-57.

[5] M.S. Arcilla, J.M. van Hattem, M.R. Haverkate, M.C.J. Bootsma, P.J.J. van Genderen, A. Goorhuis, et al. Import and spread of extended-spectrum beta-lactamase-producing Enterobacteriaceae by international travellers (COMBAT study): a prospective, multicentre cohort study. The Lancet Infectious Diseases. 2017;17(1):78-85.

[6] G. Birgand, L. Armand-Lefevre, I. Lolom, E. Ruppe, A. Andremont, J.-C. Lucet. Duration of colonization by extended-spectrum beta-lactamase-producing Enterobacteriaceae after hospital discharge. Am J Infect Control. 2013;41(5):443-7.

[7] S.B. Jørgensen, A. Søraas, A. Sundsfjord, K. Liestøl, T.M. Leegaard, P.A. Jenum. Fecal carriage of extended spectrum β-lactamase producing Escherichia coli and Klebsiella pneumoniae after urinary tract infection - A three year prospective cohort study. PLOS ONE. 2017;12(3):e0173510-e.

[8] M. Plummer, A. Stukalov, M. Denwood, M.M. Plummer. Package 'rjags'. 2018.

[9] S.P. Brooks, A. Gelman. General Methods for Monitoring Convergence of Iterative Simulations. Journal of Computational and Graphical Statistics. 1998;7(4):434-55.

[10] D.J. Spiegelhalter, N.G. Best, B.P. Carlin, A.J.J.o.t.R.S.S.S.B. Van Der Linde. Bayesian measures of model complexity and fit. 2002;64(4):583-639.
